# Foodborne illness outbreaks linked to unpasteurised milk and relationship to changes in state laws – United States, 1998–2018

**DOI:** 10.1017/S0950268822001649

**Published:** 2022-10-25

**Authors:** Lia Koski, Hannah Kisselburgh, Lisa Landsman, Rachel Hulkower, Mara Howard-Williams, Zainab Salah, Sunkyung Kim, Beau B. Bruce, Michael C. Bazaco, Michael B. Batz, Cary Chen Parker, Cynthia L. Leonard, Atin R. Datta, Elizabeth N. Williams, G. Sean Stapleton, Matthew Penn, Hilary K. Whitham, Megin Nichols

**Affiliations:** 1 Division of Foodborne, Waterborne, and Environmental Diseases, Centers for Disease Control and Prevention, Atlanta, GA, USA; 2 CAITTA, Inc., Herndon, VA, USA; 3 Public Health Law Program, Centers for Disease Control and Prevention, Atlanta, GA, USA; 4 Center for Food Safety and Applied Nutrition, Food and Drug Administration, College Park, MD, USA; 5 Oak Ridge Institute for Science and Education, Oak Ridge, TN, USA

**Keywords:** foodborne illness, outbreak, public health, unpasteurised milk

## Abstract

Consumption of unpasteurised milk in the United States has presented a public health challenge for decades because of the increased risk of pathogen transmission causing illness outbreaks. We analysed Foodborne Disease Outbreak Surveillance System data to characterise unpasteurised milk outbreaks. Using Poisson and negative binomial regression, we compared the number of outbreaks and outbreak-associated illnesses between jurisdictions grouped by legal status of unpasteurised milk sale based on a May 2019 survey of state laws. During 2013–2018, 75 outbreaks with 675 illnesses occurred that were linked to unpasteurised milk; of these, 325 illnesses (48%) were among people aged 0–19 years. Of 74 single-state outbreaks, 58 (78%) occurred in states where the sale of unpasteurised milk was expressly allowed. Compared with jurisdictions where retail sales were prohibited (*n* = 24), those where sales were expressly allowed (*n* = 27) were estimated to have 3.2 (95% CI 1.4–7.6) times greater number of outbreaks; of these, jurisdictions where sale was allowed in retail stores (*n* = 14) had 3.6 (95% CI 1.3–9.6) times greater number of outbreaks compared with those where sale was allowed on-farm only (*n* = 13). This study supports findings of previously published reports indicating that state laws resulting in increased availability of unpasteurised milk are associated with more outbreak-associated illnesses and outbreaks.

## Introduction

Consumption of unpasteurised or ‘raw’ milk can result in transmission of pathogens, such as *Campylobacter*, *Cryptosporidium*, Shiga toxin-producing *Escherichia coli* (STEC), *Listeria monocytogenes,* and *Salmonella enterica*, which can cause severe illness and death [1, 2]. In addition to acute illness, these infections can have long-term consequences, such as kidney failure resulting from haemolytic uraemic syndrome (HUS), Guillain–Barré syndrome (GBS), reactive arthritis, and functional bowel disorders [3–7]. Children aged less than 5 years, adults aged ≥65 years, people with weakened immune systems, and pregnant people and their unborn babies are at greater risk for severe outcomes or death when infected with enteric pathogens [1, 8].

In the early twentieth century, routine pasteurisation of milk from cows became widespread in the United States and led to dramatic reductions in diseases and infant mortality previously associated with unpasteurised milk consumption [9, 10]. Pasteurisation ensures that fluid milk and milk products do not contain harmful pathogens by heating every particle of milk to one of the appropriate temperatures for at least the corresponding specified lengths of time [11]. Adherence to strict sanitation practices during the milking process may help to reduce, but does not eliminate, the risk of pathogen contamination of unpasteurised milk and does not significantly reduce illnesses or outbreaks [11, 12]. Therefore, pasteurisation is essential in eliminating pathogens. Previous studies have demonstrated that consumption of unpasteurised dairy products compared with pasteurised dairy products leads to increased incidence of outbreaks, illnesses and hospitalisations. From 1993–2006, the estimated incidence of outbreaks involving unpasteurised dairy products was approximately 150 times greater per pound of dairy product consumed than the incidence involving pasteurised products [2]. In another study, unpasteurised dairy products were associated with approximately 840 times more illnesses and 45 times more hospitalisations than pasteurised products [8].

Both federal and state laws govern the sale of unpasteurised milk. In 1987, FDA mandated that all milk and milk products in final package form for direct human consumption be pasteurised before shipping in interstate commerce [13]. The sale of unpasteurised milk within each state is governed by state laws, which vary widely. A survey conducted in 2011 indicated that 20 states prohibited the sale of unpasteurised milk, but state legislation across the country has changed considerably since analysis of that survey data [14]. Legal points of sale of unpasteurised milk to consumers in the United States can include retail sales, farmers markets, and on-farm sales, depending on the jurisdiction. In addition to access via direct purchase, consumers in some states may obtain unpasteurised milk through ‘cow shares’ or ‘herd shares.’ Under these arrangements, an individual purchases an ownership interest in a cow or herd, which remains under the care of the farmer, and is thereby entitled to a portion of the unpasteurised milk produced. These arrangements have been used to attempt to circumvent state and federal law prohibitions on traditional retail sales of unpasteurised milk [15]. As a result, some states have expressly – or distinctly and explicitly stated in state law – prohibited cow or herd shares. Several states, however, have recently added laws allowing the intrastate procurement of unpasteurised milk through cow or herd shares. The federal government has deemed such transactions to be a ‘sale’ prohibited in interstate commerce by the FDA (*United States v. Allgyer,* 2012 WL 355261, at n.15 E.D. Pa. 2012). Expanding legal access to unpasteurised milk ultimately leads to increased illnesses and outbreaks [1, 2].

The prevalence of unpasteurised milk consumption in the United States has remained low, with weekly consumption estimates ranging from 1% to 2% of the United States adult population [16, 17]. Estimates of consumption for pasteurised milk in a population-based survey have been reported as high as 70.2% of the surveyed population in the week prior to interview [18]. However, outbreaks of illness linked to unpasteurised milk are disproportionately high relative to the frequency of unpasteurised milk consumption [8]. Previous studies have described the epidemiology of outbreaks linked to unpasteurised milk and milk products and the association between outbreak occurrence and state laws [1, 2]. In this analysis, we report on laws related to the sale of unpasteurised milk and utilise legal epidemiologic methods that have not been used in prior analyses pertaining to unpasteurised milk to examine the association between state laws and outbreak occurrence. Additionally, we provide a trend analysis using a Bayesian negative binomial model to examine the incidence of outbreaks and outbreak-associated illnesses linked to unpasteurised milk over time.

## Methods

### Data sources

The Foodborne Disease Outbreak Surveillance System (FDOSS) collects data from state, local, and territorial health departments and federal investigators on pathogens, foods, settings, and other characteristics of foodborne disease outbreaks (defined as ≥two similar illnesses resulting from the ingestion of a common food) submitted through CDC's National Outbreak Reporting System (NORS) [19]. We obtained data from FDOSS on 3 February 2020, to examine reported foodborne illness outbreaks during 1998–2018, including outbreaks in which the confirmed or suspected source was unpasteurised milk. For this analysis, unpasteurised milk is defined as fluid milk for human consumption that has not been pasteurised to kill harmful microorganisms. We excluded outbreaks linked to dairy products made from unpasteurised milk, such as cheeses, ice cream, and yogurt; similarly, we excluded outbreaks linked exclusively to unpasteurised chocolate milk or other flavoured milk. These other dairy products were excluded to be consistent with the intent of the legal analysis focusing exclusively on unpasteurised fluid milk.

### Trend analysis

We conducted a trend analysis using a Bayesian negative binomial model fit on number of outbreaks as the outcome with a penalised thin plate spline smoother on year using R 4.0.0 and bamlss 1.1–2 with default settings (R Foundation for Statistical Computing, Vienna, Austria) [20]. Differences in mean number of outbreaks during 1998–2018 in 7-year time intervals were calculated. We reported mean and 95% credible intervals for posterior distributions. This was repeated with outbreak-associated illness set as the outcome, and difference in mean number of outbreak-associated illness during 1998–2018 in 7-year time intervals were calculated.

### Descriptive analysis

We conducted a descriptive statistical analysis of epidemiologic variables collected for outbreaks during 2013–2018 including pathogen, patient demographics, health outcome variables and traceback investigation information for the farm where unpasteurised milk was produced. The number of primary cases reported in FDOSS was used as the number of illnesses for each outbreak.

### Legal analysis

Using legal epidemiology methods [21], we collected state statutes and regulations from 51 jurisdictions (50 states and the District of Columbia) related to the sale of unpasteurised milk for human consumption. The laws were collected and downloaded on 8 May 2019, using uniform search terms in Westlaw, a legal research database (Thomson Reuters, Eagan, Minnesota). First, we conducted a cross-sectional assessment, analysing laws that were in effect on the download date, across the 51 jurisdictions. Second, we conducted a policy surveillance study, tracking relevant amendments to those laws that were enacted from 1 January 2012, through 8 May 2019. The time period of the epidemiologic analysis included data from outbreaks during 2013–2018 to capture potential impacts of laws that were enacted in 2012 and was limited to 2018 based on availability of outbreak data at the time of analysis. Because our legal analysis was limited to laws related to the sale of unpasteurised milk for human consumption, the legal dataset did not include laws related to unpasteurised cheese or milk products or unpasteurised milk as pet food or commercial feed.

We categorised each jurisdiction's laws using the Public Health Law Information Portal (PHLIP). PHLIP is an online platform that enables users to code and track the changing text of laws and policies across jurisdictions and over time and to create scientifically rigorous datasets on discrete features of those laws and policies. PHLIP is available to researchers and their collaborators upon request. State laws were coded for whether the sale of unpasteurised milk for human consumption was allowed or prohibited and, if allowed, whether sale was legal in retail stores, on the farm where the milk was produced, or at farmers markets. We also coded the jurisdictions' laws for provisions allowing or prohibiting cow or herd shares. For coding purposes, herd shares were not considered the ‘sale’ of unpasteurised milk but instead a separate category for its acquisition. When conducting policy surveillance over time, we identified and categorised state laws that had relevant amendments during the study period to create a longitudinal dataset from 2012–2018. A second naïve reviewer coded both the cross-sectional and longitudinal data, and any divergences were resolved through a third-party validation process. The legal analysis was limited to codified state statutes and regulations and does not reflect the contribution of case law, internal policies, municipal and local laws, or implementation or enforcement of the statutory or regulatory requirements.

### Analysis of outbreaks and outbreak-associated illnesses by legal status

We fit a Poisson or negative binomial regression (depending on over-dispersion of the data) by using the number of outbreaks or outbreak-associated illnesses from 2013–2018 for each jurisdiction as the outcome variable and the legal status as the exposure variable to determine if the expected number of outbreaks or outbreak-associated illnesses would differ between jurisdictions based on legal status. Each outcome variable was analysed in three comparisons for a total of six models run in this analysis. The three comparisons performed included: (1) for all jurisdictions, jurisdictions that allowed sale of unpasteurised milk were compared to jurisdictions that prohibited sale, (2) for jurisdictions that allowed unpasteurised milk sale, jurisdictions that allowed retail sale were compared to jurisdictions that only allowed sale on-farm, and (3) for jurisdictions that prohibited unpasteurised milk sale, jurisdictions that allowed herd shares were compared to jurisdictions with no express reference to herd shares. The estimated coefficient in this analysis represents the increase in the incidence rate of the outcome (number of outbreaks or outbreak-associated illnesses) for each jurisdiction compared to the reference jurisdiction. Both the 95% confidence interval (95% CI) and the *P*-values bootstrapped standard error from 500 re-samplings to adjust for uncertainty of the estimates. We used SAS 9.4 (SAS Institute Inc., Cary, NC, USA) and Microsoft Excel to manage and analyse outbreak data and legal data.

## Results

### Data source

During 1998–2018, health departments reported 21919 foodborne outbreaks and 423 595 outbreak-associated illnesses to FDOSS. Of these, 202 outbreaks (0.9%) and 2645 illnesses (0.6%) were linked to unpasteurised milk (Table 1), including 228 hospitalisations and three deaths. During the same time period, 9 outbreaks (0.04%) and 2133 illnesses (0.5%) linked to pasteurised milk were reported, including 33 hospitalisations and three deaths.

**Table 1.** Number of outbreaks and outbreak-associated illnesses linked to unpasteurised and pasteurised milk – FDOSS, United States, 1998–2018

Year	No. of outbreaks			No. of illnesses		
	Unpasteurised milk	Pasteurised milk	All foodborne vehicles	Unpasteurised milk	Pasteurised milk	All foodborne vehicles
1998	5	0	1317	60	0	27 126
1999	1	0	1336	2	0	24 878
2000	9	1	1403	149	14	25 439
2001	3	0	1249	281	0	25 202
2002	5	1	1318	180	116	24 951
2003	3	0	1089	11	0	23 079
2004	2	1	1328	38	100	29 034
2005	10	1	964	131	200	19 900
2006	10	1	1256	75	1644^a^	28 881
2007	10	1	1098	148	5	21 302
2008	13	0	1028	148	0	23 133
2009	5	0	668	147	0	13 790
2010	18	0	853	188	0	15 865
2011	19	1	795	154	16	14 300
2012	14	1	835	258	6	15 002
2013	16	0	829	157	0	13 516
2014	15	1	874	209	32	13 343
2015	11	0	926	53	0	15 555
2016	14	0	853	116	0	14 410
2017	10	0	856	59	0	15 009
2018	9	0	1044	81	0	19 880
Total	202	9	21 919	2645	2133	423 595

a Large outbreak linked to pasteurised milk in California caused by *Campylobacter* [33].

### Trend analysis

**Fig. 1.**
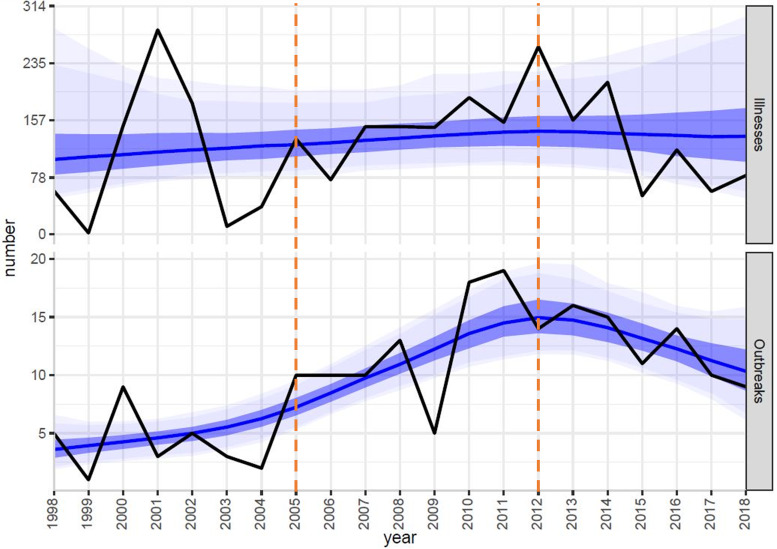
Bayesian negative binomial model (blue line) fit using a penalised thin plate spline to number of outbreak-associated illnesses (black line; top panel) and number of outbreaks (black line; bottom panel) linked to unpasteurised milk per year with 50% (dark blue), 90% (lighter blue), and 95% (lightest blue) credible intervals – FDOSS, United States, 1998–2018. This trend analysis was performed using R 4.0.0 and bamlss 1.1–2 with default settings [20]. Orange vertical lines demonstrate the 7-year time intervals compared by mean number of outbreaks and mean number of outbreak-associated illnesses.

During 1998–2018, the median number of outbreaks linked to unpasteurised milk per year during the study period was 10 (range: 1–19) outbreaks. The estimated mean number of outbreaks per year was significantly higher during 2005**–**2011 (+6.2; 95% CI +4.0 to +9.0) and 2012–2018 (+8.3; 95% CI+5.5 to +11.6) compared to 1998**–**2004, but there was no significant change in the number of outbreak-associated illnesses (Table 2; Fig. 1).

**Table 2.** The annual mean and Bayesian negative binomial model estimated change in the mean number of outbreaks and outbreak-associated illnesses linked to unpasteurised milk by 7-year periods – FDOSS, United States, 1998–2018

	No. of outbreaks per year		No. of outbreak-associated illnesses per year	
Time period	Mean	Model estimated change in mean compared to 1998–2004 (95% CI)	Mean	Model estimated change in mean compared to 1998–2004 (95% CI)
1998–2004	4	Ref	103	Ref
2005–2011	12.1	+6.2 (+4.0 to +9.0)	142	+16.5 (−71.5 to +81.6)
2012–2018	12.7	+8.3 (+5.5 to +11.6)	133	+21.3 (−112 to +158)

CI, credible interval.

Standard Bayesian negative-binomial model assumptions for trend analysis were applied [20].

### Descriptive analysis

During 2013**–**2018, there were 75 outbreaks and 675 illnesses linked to unpasteurised milk (Table 3). Among ill people with information available, 263 (39%) were female; 93 (14%) were young children aged <5 years, and 232 (34%) were aged 5–19 years. These outbreaks resulted in a reported 98 (15%) hospitalisations, including ten HUS cases and two GBS or Miller Fisher syndrome cases. Two deaths were reported. There were more outbreaks (49%) and illnesses (57%) caused by *Campylobacter* compared with other pathogens (Table 3).

**Table 3.** Unpasteurised milk associated outbreaks, illnesses and hospitalisations by pathogen – FDOSS, United States, 2013–2018

Pathogen	No. (%) of outbreaks	No. (%) of illnesses	No. (%) of hospitalisations	Percent of ill people hospitalised (%)
*Campylobacter*	37 (49)	382 (57)	31 (32)	8
*Cryptosporidium*	8 (11)	31 (5)	2 (2)	6
*Listeria monocytogenes*	1(1)	2 (0)	2 (2)	100
*Salmonella*	13 (17)	109 (16)	17 (17)	16
STEC^a^	11 (15)	71 (11)	30 (31)	42
Multiple^b^	5 (7)	80 (12)	16 (16)	20
**Total**	**75**	**675**	**98**	

a STEC, Shiga toxin-producing *Escherichia coli.*

b Seventy outbreaks were caused by a single pathogen. Among outbreaks with multiple etiologies (*n* = 5), all included *Campylobacter* and at least one other pathogen: STEC (*n* = 2), *Cryptosporidium* (*n* = 1), *Salmonella* (*n* = 1), and *Cryptosporidium* and STEC (*n* = 1).

**Fig. 2.**
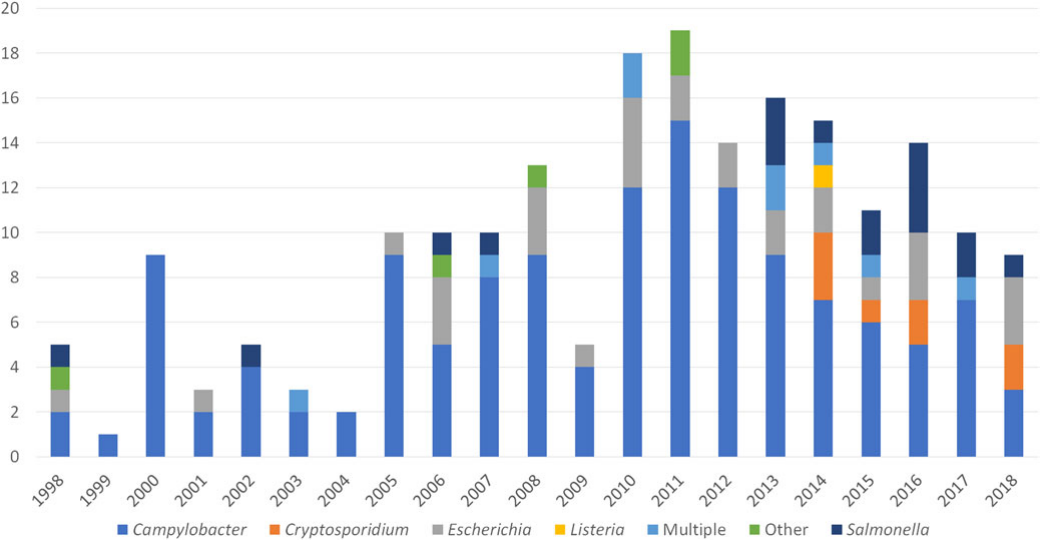
Unpasteurised milk-associated outbreaks, by year and enteric pathogen – FDOSS, United States, 1998–2018.

The number of reported outbreaks caused by *Campylobacter* decreased during 2013**–**2018 from nine outbreaks in 2013 to three in 2018 (Fig. 2). The first and only reported outbreak of *L. monocytogenes* during 1998**–**2018 included illnesses first reported in 2014. Seventy outbreaks were caused by a single pathogen. Among outbreaks with multiple aetiologies from 2013**–**2018 (*n* = 5), all included *Campylobacter* and at least one other pathogen: STEC (*n* = 2), *Cryptosporidium* (*n* = 1), *Salmonella* (*n* = 1), and *Cryptosporidium* and STEC (*n* = 1). *L. monocytogenes* and STEC outbreaks caused higher frequencies of hospitalisations among ill people (100% and 42%, respectively) compared with other pathogens (Table 3). Two deaths were reported during the study period; one resulted from *Campylobacter* and the other from *L. monocytogenes*.

Of the 75 outbreaks linked to unpasteurised milk during 2013**–**2018, 74 were single-state outbreaks and one was a multistate outbreak. The multistate listeriosis outbreak was associated with unpasteurised milk produced in Pennsylvania and unlawfully shipped in interstate commerce for direct human consumption, resulting in consumption and illnesses in Florida and California [6]. Among single-state outbreaks, Utah had the highest number of reported outbreaks (*n* = 14), followed by Pennsylvania (*n* = 9), Ohio (*n* = 7), Idaho (*n* = 5), and Texas (*n* = 4). Twenty-one states had less than four outbreaks each during 2013**–**2018 (Table 4). Traceback investigations were conducted for 11 (15%) outbreaks during this time to identify farms where the unpasteurised milk was produced; of these, four (36%) resulted in a recall of the product.

**Table 4.** State laws governing the sale of unpasteurised milk, as of 8 May 2019, with amendments as indicated for 10 states during 2012–2018 and number of outbreaks by state 2013–2018

		Retail sale allowed		Herd shares:		Sale at farmers markets:		
State	Sale prohibited in all categories	Stores, food establishments or general reference to retail sale	Sale on farm only allowed	Expressly allowed	Expressly prohibited	Expressly allowed	Expressly prohibited	No. of outbreaks during 2013–2018
Alabama	X						X	
Alaska	X			X				2
Arizona		X						
Arkansas^1^ 1/1/12–8/15/13	X							
Arkansas 8/16/13–5/8/19			X					
California		X						3
Colorado	X			X			X	3
Connecticut 1/1/12–6/22/15		X						1
Connecticut 6/23/15–5/8/19		X		X				
Delaware	X							
Florida	X							1
Georgia	X							
Hawaii	X							
Idaho		X		X				5
Illinois 1/1/12–1/28/16			X					
Illinois 1/29/16–5/8/19			X	X				2
Indiana	X							
Iowa	X							
Kansas			X					
Kentucky	X							1
Louisiana	X				X			
Maine		X						1
Maryland	X							
Massachusetts		X						2
Michigan	X							3
Minnesota			X					2
Mississippi	X							
Missouri			X					1
Montana	X							1
Nebraska			X					
Nevada		X			X			
New Hampshire		X				X		1
New Jersey	X							1
New Mexico		X						1
New York			X					1
North Carolina 1/1/12–9/30/18	X				X			
North Carolina 10/1/18–5/8/19	X			X				
North Dakota 1/1/12–7/31/13	X							
North Dakota 8/1/13–5/8/19	X			X				
Ohio		X						7
Oklahoma			X					
Oregon			X					
Pennsylvania		X						9
Rhode Island	X							
South Carolina		X				X		
South Dakota^2^ 1/1/12–6/30/15			X					
South Dakota 7/1/15–5/8/19			X				X	
Tennessee	X			X				3
Texas			X				X	4
Utah^3^ 1/1/12–5/11/15		X			X			5
Utah 5/12/15–5/8/19		X		X				9
Vermont 1/1/12–6/30/14			X					
Vermont 7/1/14–5/8/19			X			X		
Virginia	X							1
Washington		X		X				2
Washington, D.C.	X							
West Virginia 1/1/12–5/22/16	X				X			
West Virginia 5/23/16–5/8/19	X			X				
Wisconsin			X					2
Wyoming 1/1/12–12/9/12	X							
Wyoming 12/10/12–5/8/19	X			X				
Totals (State totals 5/8/19)	24	14	13	12	2	3	4	74^a^

**Provisions reviewed**: Ala. Admin. Code r. 420-3-16-0.12; Ala. Admin. Code r. 80-7-1-0.04(9)(b); Ala. Admin. Code r. 350-X-1-0.05(9)(a); Alaska Admin. Code tit. 18, §§ 32.010(c), 32.020, 32.060, 32.215; Ariz. Rev. Stat. Ann. § 3-606(A)(1); Ark. Code Ann. § 20-59-248; Code Ark. R. 007.10.17-2, 007.10.31-2; Cal. Food & Agric. Code §§ 35 861(e), 35 891(c), 35 928(a), (f) (West); Cal. Health & Safety Code §§ 113789, 114024 (West); Cal. Code Regs. tit. 17, § 11 380(d); Colo. Rev. Stat. Ann. § 25-5.5-117(1), (3) (West); Conn. Gen. Stat. Ann. §§ 22-129, 22-172(b), 22-173a (West); 16 Del. Admin. Code § 4461-2.0; Fla. Stat. Ann. § 502.091(1) (West); Ga. Code Ann. § 26-2-238 (West); Ga. Comp. R. & Regs. 40-2-1-0.01(a), 40-2-15-0.01; Haw. Code R. § 11-15-46 (West); Idaho Code Ann. § 37-1101 (West); Idaho Admin. Code r. 02.04.13.009(02), 02.04.13.020, 02.04.13.030, 02.04.13.040(06); 410 Ill. Comp. Stat. Ann. 635/8 (West); Ill. Admin. Code tit. 77, §§ 775.10, 775.50, 775.55; Ind. Code Ann. §§ 15-18-1-20, 15-18-1-21 (West); 345 Ind. Admin. Code 8-2-1.9; Iowa Code Ann. § 192.103 (West); Kan. Stat. Ann. §§ 65-771, 65-773, 65-789(d) (West); 902 Ky Admin Regs. 50:110; La. Admin. Code tit. 51, Pt VII, § 323(A), (E); Me. Rev. Stat. Ann. tit. 7, §§ 2902-B, 2910; Code Me. R. tit. 01-100, Ch. 329, § V; Md. Code Ann., Health-General § 21-434 (West); Md. Code Regs. 10.15.06.12; Mass. Gen. Laws Ann. ch. 94 § 13 (West); 330 Mass. Code Regs. 27.01, 27.02, 27.06-08; Mich. Comp. Laws Ann. §§ 288.538(1), 288.696(1) (West); Minn. Stat. Ann. § 32D.20(1) (West); Miss. Code Ann. § 75-31-65 (West); 15 Code Miss. R. Pt. 13, Subpt. 74, R. 2.1; Mo. Ann. Stat. § 196.935; Mont. Admin. R. 32.8.102-103, 37.110.311; Neb. Rev. Stat. Ann. § 2-3969(3) (West); Nev. Rev. Stat. Ann. §§ 584.207(2), 584.208(1); Nev. Admin. Code §§ 584.2021, 584.2855; N.H. Rev. Stat. Ann. §§ 184:30-a, 184:84(V); N.H. Code Admin. R. MIL 301.03; N.J. Stat. Ann. § 24:10-57.17 (West); N.J. Admin. Code § 8:24-3.2(i)(2); N.M. Stat. Ann. § 25-8-1 (West); N.M. Admin. Code 21.34.2.6; N.Y. Comp. Codes R. & Regs. tit. 1, §§ 2.2(pp), 2.3; N.C. Gen. Stat. Ann. § 106-266.35 (West); N.D. Cent. Code Ann. §§ 4.1-25-01(29), 4.1-25-30, 4.1-25-40, 4.1-25-51 (West); Ohio Rev. Code Ann. § 917.04 (West); Ohio Admin. Code 901:11-1-04(A), (E); Okla. Stat. Ann. tit. 2, §§ 7-406, 7-414(A)(1); Or. Rev. Stat. Ann. §§ 621-012, 621-116, 621-117 (West); 7 Pa. Code §§ 59a.12(b)(3), 59a.402(b), 59a.410(a), (b); 21 R.I. Gen. Laws Ann. § 21-2-2(8) (West); S.C. Code Ann. Regs. 61-25 §§ 1-201.10(B)(1)(96), 3-2, 3-202.14(B), (E), 3-603.11(D), 9-11; S.C. Code Ann. Regs. 61-34 § III(C); S.D. Codified Laws § 39-6-3; S.D. Admin. R. 12:05:07:10, 44:02:07:16(1); Tenn. Code Ann. § 53-3-119 (West); Tenn. Comp. R. & Regs. 0080-03-02-0.11, 1200-23-01-0.03(2)(b)(4)(ii)(I); Tex. Health & Safety Code Ann. § 437.020(e) (West); 25 Tex. Admin. Code §§ 217.1, 217.22, 217.32, 228.2, 228.63, 229.701; Utah Code Ann. §§ 4-3-102(2), 4-3-502(2)(a), §4-3-503 (West); Utah Admin. Code r. 70-330-5(7), 70-330-9(1); Vt. Stat. Ann. tit. 6, §§ 2775, 2777, 2778(b)(2)(B) (West); 2 Va. Admin. Code §§ 5-490-30, 5-490-70(A), 5-490-73, 5-490-75; Wash. Rev. Code Ann. § 15.36.012; Wash. Admin. Code §§ 246-215-03250, 246-215- 03 610; D.C. Mun. Regs. tit. 16, § 3620; D.C. Mun. Regs. tit. 25-A, § 702; W. Va. Code Ann. § 19-1-7 (West); W. Va. Code R. § 64-34-2; Wis. Stat. Ann. § 97.24 (West); Wis. Admin. Code ATCP §§ 65.52, 75.3-214; Wyo. Admin. Code 010.0003.3 § 8.

a One multistate outbreak linked to unpasteurised milk is excluded from this table. ^1^Blue color represents states with a change in the law during the study period; sale/distribution previously more restricted. Effective dates are indicated, with arbitrary initial date of 1/1/12 and cut-off date of 5/8/19 (date of download of laws for analysis). ^2^Orange color represents state with change in law; previously less restrictive. Effective dates are indicated, with arbitrary initial date of January 1, 2012, and cut-off date of May 8, 2019 (date of download of laws for analysis). ^3^Retail sale allowed from producer-owned retail store.

### Legal analysis

**Fig. 3.**
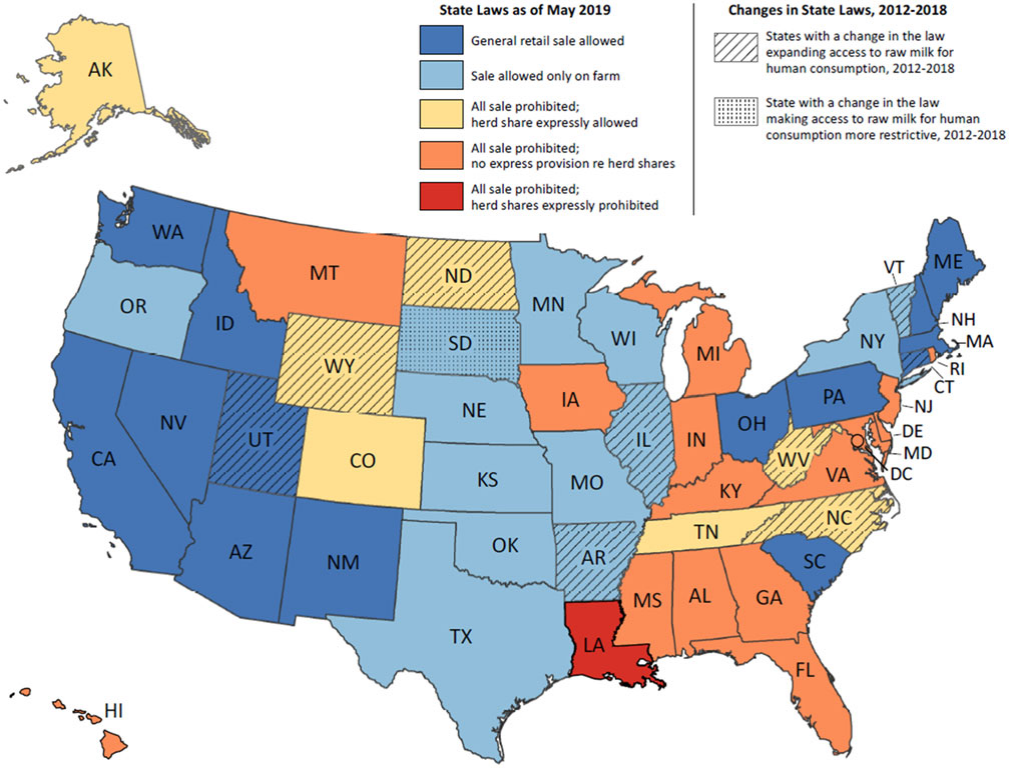
Laws related to the sale of unpasteurised milk as of May 2019 and changes to laws 2012–2018 – United States. Provisions reviewed to generate this map are referenced in Table 4.

As of May 2019, 24 jurisdictions (23 states and D.C.) prohibited all sale of unpasteurised milk for human consumption, and 27 states allowed the sale of unpasteurised milk (Table 4; Fig. 3). Of the 27 states allowing sale, 14 allowed general retail sale, which included provisions allowing for retail sale without specifying or restricting the location, or sale at stores (e.g., grocery stores) or other ‘food establishments.’ The remaining 13 states restricted sale to the farm where the milk was produced. Five states had laws specifically referencing sale of unpasteurised milk at farmers markets; three expressly allowed sale, and two expressly prohibited such sale. Most jurisdictions (*n* = 46) had no specific reference to sale at farmers markets.

Among all jurisdictions, 14 states had express provisions concerning herd shares; of these, two states expressly prohibited herd shares, and 12 states expressly allowed them. Among the 12 states expressly allowing herd shares, seven states prohibited all other sale of unpasteurised milk. Only one state, Louisiana, expressly prohibited sale of unpasteurised milk and expressly prohibited herd shares.

During the period 1 January 2012, through 8 May 2019, 10 states had a relevant amendment to a law related to the sale of unpasteurised milk for human consumption (Fig. 3). The most recent amendment was enacted as of 1 October 2018, in North Carolina (Table 4). Nine of these 10 states expanded legal access to unpasteurised milk. Arkansas shifted from all sale prohibited to sale allowed on the farm where produced. In Vermont, where sale was previously restricted to the farm where produced, a new provision was added expressly allowing sale at farmers markets. Seven states amended laws regarding herd shares, including a change from expressly prohibiting herd shares to allowing them (North Carolina, Utah and West Virginia) or from no specific reference to herd shares to an express provision allowing them (Connecticut, Illinois, North Dakota and Wyoming). Only one state amended its law to restrict the sale of unpasteurised milk: South Dakota added an express prohibition on the sale of unpasteurised milk at farmers markets. Earlier versions of the South Dakota law did not specifically reference sale at farmers markets, although they did allow direct delivery to consumers from the farm where milk was produced.

Of the 74 single-state outbreaks linked to unpasteurised milk during 2013**–**2018, 58 (78%) outbreaks occurred in states where the sale of unpasteurised milk was expressly allowed in retail stores, at farmers markets, or on the farm where produced. Eight (11%) outbreaks occurred in states where retail sale was prohibited but acquisition of unpasteurised milk was expressly allowed through herd shares; and eight (11%) outbreaks occurred in states where the sale of unpasteurised milk was prohibited by state law and there was no specific provision about herd shares. Outbreaks occurred in three of the 10 states where there was change in the governing law during 2012–2018: one outbreak occurred in Connecticut before the amendment of the law, two outbreaks in Illinois occurred after amendment of the law, and Utah reported five outbreaks before and nine outbreaks after amendment of the law (Table 4). There were no outbreaks reported before or after the amendment of the law in Arkansas, North Carolina, North Dakota, South Dakota, Vermont, West Virginia, or Wyoming (Table 4). Of the 25 total jurisdictions (24 states and D.C.) that had no reported outbreaks during 2013**–**2018, 15 (60%) were jurisdictions where all sale was prohibited (although one expressly allowed herd shares during the entire six-year period and three expanded access to allow herd shares during that time), seven (28%) were states where sale was allowed only on the farm where produced, and three (12%) were states that allowed general retail sale. No outbreaks were reported in Louisiana, where the sale of unpasteurised milk and herd shares were expressly prohibited (Table 4).

### Analysis of outbreaks and outbreak-associated illnesses by legal status

Jurisdictions where unpasteurised milk sales were allowed were estimated to have 3.2 (95% CI 1.4–7.6) times greater number of outbreaks and 2.9 (95% CI 0.8–10) times greater number of outbreak-associated illnesses compared with jurisdictions where retail sales were prohibited (Table 5). Among jurisdictions where sale of unpasteurised milk was allowed (*n* = 27), those where sale was allowed in retail stores were estimated to have 3.6 (95% CI 1.3–9.6) times greater number of outbreaks and 3.2 (95% CI 0.8–13.3) times greater number of outbreak-associated illnesses compared with jurisdictions where sale was allowed on farm only. Finally, among jurisdictions where sale of unpasteurised milk was prohibited (*n* = 23), the expected number of outbreaks for those that had no express reference to herd shares was not statistically different from those jurisdictions where herd shares were expressly allowed (incidence rate ratio (IRR): 2.3, 95% CI 0.9–6.0). Similarly, the number of outbreak-associated illnesses among jurisdictions where sale of unpasteurised milk was prohibited was similar to the number of outbreak-associated illnesses for those that had no express reference to herd shares (IRR: 6.0; 95% CI 0.7–51.1)

**Table 5.** The number of jurisdictions, outbreaks, outbreak associated-illnesses, and the IRRs of expected number of outbreaks and illnesses, by legal status for unpasteurised milk sale, FDOSS, United States, 2013–2018

		Outbreaks Models			Outbreak-Associated Illnesses Models		
Models for legality of sales and types of sales	No. (%) of jurisdictions	No. (%) of outbreaks^a^	IRR (95% CI)^b^	*P*-value^b^	No. (%) of illnesses	IRR (95% CI)^b^	*P*-value^b^
Models for all jurisdictions (*n* = 51)							
Sale allowed^c^^,^^d^	27 (53)	58 (78)	3.2^e^ (1.4–7.6)	0.006	514 (76)	2.9^e^ (0.8–10.0)	0.086
Sale prohibited	24 (47)	16 (22)	Ref	–	159 (24)	Ref	
Models for jurisdictions that allowed sales (*n* = 27)							
Retail sale allowed	14 (52)	46 (79)	3.6^e^ (1.3–9.6)	0.012	398 (77)	3.2^e^ (0.8–13.3)	0.102
Sale on farm only	13 (48)	12 (21)	Ref		116 (23)	Ref	
Models for jurisdictions that prohibited sales (*n* = 23)^f^							
Herd shares allowed	7 (30)	8 (50)	2.3^g^ (0.9–6.0)	0.084	115 (72)	6.0^e^ (0.7–51.1)	0.098
No express reference to herd shares	16 (70)	8 (50)	Ref		44 (28)	Ref	

a Total outbreak number (*n* = 74) does not include one multistate outbreak.

b Both the 95% CI and the *P*-values bootstrapped standard error from 500 re-samplings to adjust for uncertainty of the estimates.

c Sale allowed includes retail sales and/or on-farm only sales.

d Arkansas, which had a change in the relevant law during the study period, was considered as a jurisdiction that allowed sale.

e Negative binomial regression.

f Louisiana, where retail sale and herd shares are both expressly prohibited, was not included in this analysis. No outbreaks occurred in this jurisdiction.

g Poisson regression.

## Discussion

Our findings support those from previous studies that access to unpasteurised milk leads to more outbreaks than pasteurised milk despite far less consumption of unpasteurised milk by the population [1, 2, 8, 18]. This has continued to be a public health challenge in the United States despite persistent recommendations against the practice [1, 2]. Although the mean number of outbreaks has increased over time, the mean number of illnesses per outbreak (outbreak size) has not increased. This might be a result of laws that prohibit interstate sale and distribution of unpasteurised fluid milk for direct human consumption. This could also be influenced by changes in outbreak detection and response over time. Furthermore, prohibiting interstate sale might limit outbreak size only to those with access, and expansion of sale beyond state lines could increase the number of outbreaks and outbreak size. Outbreaks linked to unpasteurised milk in this analysis disproportionately affected younger people, with over half of illnesses occurring in people aged 19 years and younger. Children are at greater risk for severe infection and might not make dietary decisions for themselves. Educating parents and caregivers about risks of outbreak-associated illnesses linked to unpasteurised milk consumption among children may help prevent consumption and reduce illnesses among this population.

In our analysis, *Campylobacter* was the pathogen responsible for the most reported outbreaks and outbreak-associated illnesses linked to unpasteurised milk consumption and one of two reported deaths. The number of reported *Campylobacter* outbreaks linked to unpasteurised milk decreased during 2013**–**2018. Additional data are required to determine if this indicates a true and consistent decline in the number of outbreaks associated with unpasteurised milk or if this is an artefact of under-reporting. Prior to 2020, studies have reported an increase in the incidence of foodborne *Campylobacter*-associated outbreaks and illnesses in the United States [22–24], and unpasteurised dairy is among the most common vehicles for these outbreaks and illnesses [22, 23]. As such, continued surveillance will be important for understanding how the number and characteristics of outbreaks associated with unpasteurised milk changes over time.

Outbreaks linked to unpasteurised milk can be difficult to identify and trace to a production source. As part of unpasteurised milk sales agreements (such as herd shares), customers may not report product consumption and may withhold information about the source of the milk, which prevents traceback and implementation of farm-level interventions [25]. In this analysis, traceback information was available for a small fraction of the outbreaks linked to unpasteurised milk; however, when traceback activities were conducted based on available information, 36% resulted in product recalls. Recalls can remove contaminated food products from circulation, reduce illnesses, and stop outbreaks. Traceback to farms is critical to conducting case finding through purchase records or customer lists and implementing disease prevention measures such as basic sanitation, hygiene, and education regarding potential routes of milk contamination. Without traceback, ability to prevent additional illnesses or stop outbreaks might be limited.

In our analysis of outbreaks based on state laws, 78% of outbreaks linked to unpasteurised milk occurred in states where the sale of unpasteurised milk was expressly allowed in retail stores, on farms and/or in farmers markets, or in states that expressly allowed herd shares. This finding is consistent with findings from other studies [1, 2]. We also found that among states where sale of unpasteurised milk was allowed, those that allowed retail sale had significantly more outbreaks than states where only on-farm sale was allowed. This suggests that greater access to unpasteurised milk through retail sale increases outbreak occurrence.

Outbreaks were also reported in states that prohibited the sale of unpasteurised milk. Possible explanations for this include: the state law neither expressly prohibited nor expressly allowed herd shares, people consumed unpasteurised milk labelled and sold as pet food, there were illegal sales of unpasteurised milk occurring in these states, or individual consumers crossed state borders to purchase unpasteurised milk in states where sale is legal [1, 26]. Another explanation is the recent increasing availability of online purchasing options [6]. Although prohibited under federal law, these types of online sales are not generally addressed by state laws [13]. Online platforms are one method individuals report using to obtain unpasteurised milk [6]. During outbreak investigations, it is important to include questions about online platforms when conducting traceback for potential purchase locations.

Our legal analysis identified nine states where laws were amended to increase access to unpasteurised milk, either by allowing sale where it was formerly prohibited or by expressly allowing herd shares. In two states, Utah and Illinois, where laws changed to expressly allow herd shares, there was an increase in reported outbreaks after these amendments were made (Table 4). It is important to monitor changes in states' laws and identify states that expand legal access to unpasteurised milk. In these states, public health officials might anticipate an increase in outbreaks and subsequent increases in the costs associated with investigations. Public health officials, in collaboration with physicians, veterinarians and agriculture officials, should increase awareness of all aspects of the health and economic impacts of more permissive sale of unpasteurised milk.

During the 1998–2018 timeframe when the trend analysis was conducted in this study, changes occurred in the process of outbreak reporting including the establishment of NORS in 2009 [27]. Collection of additional data elements through NORS has improved the ability to describe and prevent foodborne disease outbreaks at national and state levels through the collection of detailed information about the foods and pathogens that pose the biggest risk to human health [27]. These reporting changes can introduce surveillance artefacts that we might not be able to account for in our analysis. These may include changes in reported mode of transmission classification (foodborne to another mode), which have been demonstrated in other studies [27]. Furthermore, NORS is a voluntary reporting system, and there might be variability among jurisdictions investigating and reporting illness outbreaks, which could influence the numbers of outbreaks identified and reported for each state [28]. Additionally, most illnesses arise sporadically rather than as part of recognised outbreaks and are less likely to be attributed to a specific food or other exposure, thus potentially underestimating the true burden of disease linked to unpasteurised milk and other food vehicles [29, 30]. Finally, our analyses comparing outbreak number and outbreak-associated illnesses between states based on legal status did not control for true population at risk (i.e., those who drink unpasteurised milk) within each jurisdiction because this information is not collected through NORS. Contextualising the number of outbreaks and outbreak-associated illnesses with the total number of consumers at risk in each state could help assess if the observed rates of illness are influenced by the size of the exposed populations and not merely the legal status of the states in which they reside. Additional research evaluating the total number of servings consumed by consumers in a given time period would also enhance analysis of risk, though these data are not currently uniformly collected.

The consumption of unpasteurised milk has been connected to sporadic illnesses caused by less common pathogens such as. *Brucella* spp. and *Coxiella burnetii* [31, 32]. Given the severity and long-term health impacts of pathogens such as *Brucella* and *Coxiella burnetii,* future analyses might consider assessing the additional impact of sporadic illness associated with consumption of unpasteurised milk. In addition to maintaining surveillance activities for outbreaks linked to unpasteurised milk, it is important that surveillance and outbreak reporting systems monitor for pathogens not previously known to be transmitted through unpasteurised milk and for antimicrobial resistant pathogens.

Our legal analysis of changes to state laws during 2012–2018 has at least two intrinsic limitations. First, only laws identified in the original cross-sectional assessment were reviewed for amendments made during the study period; other relevant laws predating the 8 May 2019 collection date, but no longer in effect as of that date, may be relevant. Second, the availability of historical amendments to state regulations varies across jurisdictions, hampering uniform comparisons. We also note that, while outside the scope of this study, many state laws contain detailed requirements for licensure, permitting, certification, labelling, or marketing unpasteurised milk products. Future examination of these parameters may be useful in further establishing an association between state laws on unpasteurised milk and foodborne disease outbreaks.

In conclusion, we find that access to unpasteurised milk continues to result in outbreaks in the United States. Most outbreaks occurred in states where the sale of unpasteurised milk is expressly allowed by law, and children were more affected compared with other age groups. Pasteurisation is a critical step in eliminating pathogen contamination of milk, even when good sanitation practices are in place. Continued partnership between state and federal government is needed to coordinate outbreak investigation activities, to report outbreaks linked to unpasteurised milk, and to monitor for changes in state policy that may impact public health. Finally, in states that are considering expanding legal access, especially retail access, to unpasteurised milk there is a need for enhanced education and awareness regarding potential increases in illnesses and the resulting burden and costs for public health systems.

## Data Availability

The data that support the findings of this study are publicly available from the Centers of Disease Control and Prevention NORS website: https://wwwn.cdc.gov/norsdashboard/. Requests for data for analysis purposes may be directed to: NORSDashboard@cdc.gov.

## Footnotes

1 Blue colour represents states with a change in the law during the study period; sale/distribution previously more restricted. Effective dates are indicated, with arbitrary initial date of 1/1/12 and cut-off date of 5/8/19 (date of download of laws for analysis).

2 Orange colour represents state with change in law; previously less restrictive. Effective dates are indicated, with arbitrary initial date of 1 January 2012, and cut-off date of 8 May 2019 (date of download of laws for analysis).

3 Retail sale allowed from producer-owned retail store.
